# BREAST CANCER METASTASIS IN THE STOMACH: WHEN THE GASTRECTOMY IS INDICATED ?

**DOI:** 10.1590/0102-6720201600020005

**Published:** 2016

**Authors:** Marcus Vinicius Rozo RODRIGUES, Valdir TERCIOTI-JUNIOR, Luiz Roberto LOPES, João de Souza COELHO-NETO, Nelson Adami ANDREOLLO

**Affiliations:** Discipline of Diseases of the Digestive System and Gastrocentro Unit, Department of Surgery, Faculty of Medical Sciences, State University of Campinas - Unicamp, Campinas, SP, Brazil.

**Keywords:** Breast Neoplasms, Neoplasm Metastasis, Stomach, Drug therapy, Gastrectomy

## Abstract

**Background:**

Breast cancer is the most common malignant neoplasm in the female population. However, stomach is a rare site for metastasis, and can show up many years after initial diagnosis and treatment of the primary tumor.

**Aim:**

Analyze a case series of this tumor and propose measures that can diagnose it with more precocity.

**Methods:**

Were analyzed 12 patients with secondary gastric tumors. Immunohistochemistry has demonstrated that primary tumor was breast cancer. We retrieved information of age, histological type, interval between diagnosis of the primary breast cancer and its metastases, immunohistochemistry results, treatment and survival.

**Results:**

The mean age was 71.3 years (ranging 40-86). Ten cases had already been underwent mastectomy in the moment of the diagnosis of gastric metastasis. Two patients had diagnosis of both primary and secondary tumors concomitantly. At average, diagnosis of gastric metastasis was seven years after diagnosis of primary breast cancer (ranging 0-13). Besides, nine cases had also metastases in other organs, being bones the most affected ones. Immunohistochemistry of the metastases has shown positivity for CK7 antibody in 83.34%, estrogen receptor in 91.67%, progesterone receptor in 66.67% and AE1AE3 antibody in 75%, considering all 12 cases. Moreover, CK20 was absent significantly (66.67%). The positivity of BRST2 marker did not present statistical significance (41.67%). Eight cases were treated with chemotherapy associated or not with hormonal blockade. Surgical treatment of gastric metastasis was performed in four cases: three of them with total gastrectomy and one with distal gastrectomy. Follow-up has shown a mean survival of 14.58 months after diagnosis of metastasis, with only two patients still alive.

**Conclusion:**

Patients with a history of breast cancer presenting endoscopic diagnosis of gastric cancer it is necessary to consider the possibility of gastric metastasis of breast cancer. The confirmation is by immunohistochemistry and gastrectomy should be oriented in the absence of other secondary involvement and control of the primary lesion.

## RESUMO

**Racional:**

A neoplasia de mama é o tumor maligno mais comum na população feminina tendo o trato gastrointestinal, e mais especificamente o estômago, como local incomum para metástases.

**Objetivo:**

Analisar uma série de casos com esse tumor e propor medidas que possam diagnosticá-lo com maior precocidade.

**Métodos:**

Foram analisados retrospectivamente 12 pacientes com diagnóstico de neoplasia gástrica secundária a câncer de mama, confirmado por biópsia e imunoistoquímica. Foram analisados idade do diagnóstico, tipo histológico do tumor primário, intervalo de tempo entre o diagnóstico do tumor e a metástase, tratamento e sobrevida.

**Resultados:**

A idade média foi de 71,3 anos (40-86 anos). Em média, o diagnóstico da metástase gástrica foi de sete anos após o diagnóstico da lesão primaria (0-13 anos). Nove casos tiveram metástases em outros órgãos, sendo os ossos os locais mais acometidos. Evidenciou-se positividade de anticorpo CK7 em 90,9% casos, receptor de estrógeno em 91,67%, receptor de progesterona em 66,67% e BRST2 em 41,67%. A ausência de CK20 foi de 88,89%. Oito casos foram tratados com quimioterapia associada ou não ao bloqueio hormonal e em quatro foi indicada ressecção cirúrgica sendo em três gastrectomia total e em um caso gastrectomia subtotal. A sobrevida média foi de 14,58 meses.

**Conclusões:**

Em doentes com história prévia de câncer de mama apresentando diagnóstico endoscópico de neoplasia gástrica, é necessário considerar a possibilidade de metástase. A confirmação é feita por estudo imunoistoquímico e a gastrectomia deve ser orientada diante da ausência de outros locais de acometimento secundário e controle da lesão primária.

## INTRODUCTION

Breast cancer is the most common malignant tumor in the female population, accounting for high morbidity and mortality worldwide. In addition, metastases of breast cancer usually are directed to bones, lung, liver and brain. However, metastases to the stomach are very unusual and there are a few studies on this subject^2^ ^,^ ^6^ ^,^ ^8^ ^,^ ^9^ ^,^ ^11^ ^,^ ^12^ ^,^ ^13^ ^,^ ^17^ ^,^ ^20^. When these metastases occur, diagnostic confirmation based on immunohistochemistry is needed ^2^ ^,^ ^3^ ^,^ ^5^ ^,^ ^7^ ^,^ ^11^ ^,^ ^14^ ^,^ ^16^ ^,^ ^20^.

Considering that there are a few number of cases described in the medical literature, this paper aims to report a case series, helping to improve knowledge concerning this uncommon breast cancer metastasis.

## METHODS

This study is a non-randomized retrospective review of 12 patients with breast cancer metastasis in their stomachs that were treated from 2001 to 2011. 

Were reviewed the medical records and features considered were: age, histology, time gap between diagnosis of the primary tumor and its metastasis, hormonal receptors, histological markers BRST2, CK7, RE, RP, CK20, HER2, others organs with metastasis, upper endoscopy findings, computerized tomography scan findings, treatment and follow-up. 

A pathologist with breast cancer expertise provided data concerning histology and immunohistochemistry. Besides, treatment employed was according to guidelines established by the Brazil Ministry of Health and by the World Health Organization.

Global survival was the time gap between metastasis diagnoses until death caused by any reason. The follow-up was until March 2015, when this study ended.

A level of significance of 5% (p<0.05) was adopted.

## RESULTS

Patients in the moment of the diagnoses of the gastric metastasis were 40 to 86 years old (mean 71.3). Ten of twelve patients had been submitted to mastectomy previously and two of them had the diagnoses of gastric metastasis concomitantly with the diagnoses of the primary tumor. Thus, the time gap between primary tumor and metastasis diagnoses ranged from 0 to 13 years (average 6.75).

Ten of twelve patients with metastasis in the stomach had other organs involved at the same moment, being bones the most affected ones (nine of 12 cases, 75%). After bone involvement, were observed metastases in lungs (3/12 cases, 25%), large bowel (n=1), liver (n=1), esophagus (n=2), mediastinum (n=1) and skin (n=1).

The predominating symptoms were nausea and vomiting (n=5, 41.6%), weight loss (n=4, 33.3%), upper abdominal pain (n=3, 25%), gastric emptying impaired (16.6%) and dyspepsia (n=1, 8.3%).

Histology of the primary tumor consisted of lobular pattern in five cases (41.6%) and ductal pattern in seven (58.3%). There was not a higher mortality linked to any pattern (p=0.813).

Considering hormonal receptors, estrogen receptor was present in 11 cases (91.6%) and progesterone receptor in six (66.6%). However, estrogen receptor was searched in all cases and progesterone receptor was searched in only nine.

Considering antibodies, CK20 monoclonal antibody was absent in eight of nine cases (88.8%), CK7 antibody was present in 10 of 11 cases (90.9%), BRST2 antibody was present in only five (41.6%) although BRST2 antibody was searched in all 12 cases. None of these markers has shown an increase in mortality (p>0.05%). Finally, HER2 was searched in only three cases, being positive in two (66.6%).

**FIGURE 1:**
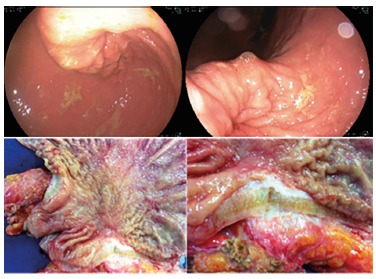
Upper endoscopy showing breast cancer metastasis to the stomach and surgical specimens showing breast cancer metastasis to the stomach like linitis

**FIGURE 2:**
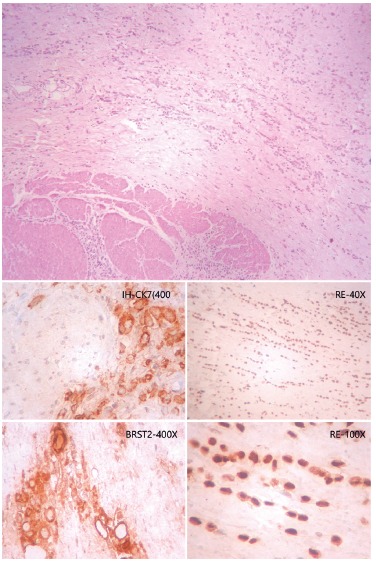
On the left, is shown neoplastic cells above the muscular layer of the stomach (HE stain, 40x augmentation). On the right, the smaller photomicrographs show positivity to immunohistochemistry markers to breast cancer (clockwise, CK7, estrogen receptor, BRST2).

Concerning treatment, eight cases received chemotherapy associated or not with hormonal blockade. Four had their metastasis in the stomach treated surgically, three of them submitted to total gastrectomy and one to distal gastrectomy. The gastrectomies were associated to a D2 level lymphadenectomy (Figures 1 and 2).

Mean survival after the diagnoses of metastasis in the stomach was only 14.58 months. However, mean survival increases to 38 months when considering only the four patients treated surgically.

## DISCUSSION

Prevalence of metastasis of a primary breast cancer to the digestive tract is rare, being calculated an occurrence of non-greater than 0.3% in the stomach resections. Nonetheless, studies of necropsies observed this uncommon event in patients with a breast cancer history ranging from 4% to 35%. Furthermore, 94% of these patients had other organs affected concomitantly ^1^ ^,^ ^6^ ^,^ ^11^.

Some authors report an average time between primary tumor diagnoses and metastasis to the stomach ranging from 4 to 10 years ^3,6,^. The mean age of these metastatic tumors is predominantly in the perimenopausal period due to hormonal imbalances typical of this phase ^10^.

The most common aspect of presentation of these metastases in the stomach is linitis, affecting muscle layer and submucosae at maximum rate of 73% of cases ^1^ ^,^ ^3^ ^,^ ^4^ ^,^ ^5^ ^,^ ^7^.

According to some authors, lobular carcinoma is the most common source of metastases to the stomach at a maximum rate of 83% of the cases ^8^ ^,^ ^15^ ^,^ ^17^. In addition, even when mix ductal-lobular carcinomas are present, it is observed a predominant component of lobular carcinoma of the metastases to the digestive tract ^2^ ^,^ ^3^.

Besides, medical literature presents us that the most frequent symptoms are dyspepsia, loss of appetite, upper abdominal pain, nausea, vomiting and gastric emptying impaired ^1^ ^,^ ^6^ ^,^ ^13^ ^,^ ^20^. Nonetheless, these symptoms lack any specificity considering that patients usually are receiving chemotherapy, radiotherapy or even suffering of electrolytic disorders. Because of that, there could be a delay in diagnoses^4^.

Subsidiary examinations like upper endoscopy, computerized tomography scans or positron emission tomography must be part of the diagnostic effort. However, they have lower specificity ^6^ ^,^ ^7^ ^,^ ^8^ ^,^ ^9^ ^,^ ^15^ ^,^ ^20^.

Finally, immunohistochemistry searching for hormonal receptors (estrogen and progesterone) show a higher rate of diagnostic evidence^1^ ^,^ ^14^.

According to medical literature, Gross cystic disease fluid protein-15 monoclonal antibody (GCDFP-15) or BRST2 has shown sensibility of 55-76% and specificity of 95-100% for the diagnosis of metastatic breast cancer ^6^ ^,^ ^9^ ^,^ ^13^. The CK7 monoclonal antibody is present in tumors with glandular pattern, being observed at a maximum rate of 90% of breast cancers. It suggests breast cancer metastasis considering that only 50-64% of adenocarcinomas of the stomach present this molecule^8^ ^,^ ^10^ ^,^ ^11^. On the other hand, CK20 antibody presence favors the diagnoses of primary cancers from stomach, large bowel and pancreas ^7^ ^,^ ^10^ ^,^ ^13^ ^,^ ^15^ ^,^ ^19^. In this study, was verified an endorsement of this immunohistochemistry profile that means positivity for estrogen receptor, progesterone receptor and CK7 antibody and negativity for CK20 antibody.

HER2 marker is present in about 15% to 20% of cases of breast cancer. Although it is not useful for diagnoses when used as an isolated marker because it is also common in primary gastric cancer, its presence infers a greater aggressiveness and a worst prognosis of the disease ^14^ ^,^ ^15^. 

There is a consensus in the literature that first line therapy for breast cancer metastasis to the stomach is chemotherapy associated or not with hormonal blockade^5^ ^,^ ^6^ ^,^ ^9^ ^,^ ^12^ ^,^ ^13^. Mean survival after diagnoses of breast cancer metastasis to the stomach was of seven months (0 to 41 months) in the absence of complications ^2^ ^,^ ^17^.

Despite the publications showing increase in survival of patients submitted to metastasis resection of liver and of the lung, there are not studies with significant evidence of these same results when metastasis is in the digestive tract^15^. However, if there is an isolated metastasis in the stomach and, concomitantly, primary tumor is controlled, it is possible to achieve an increase in survival from nine months to 44 months when gastric resection is performed ^7^ ^,^ ^12^ ^,^ ^13^ ^,^ ^15^. In this study, when was considered survival of patients submitted to gastric resection, was found out a 38 months survival, much better of the 14.38 months survival of the whole group.

Finally, gastric adenocarcinoma has a high incidence in the population, being more frequent in males ^21^. However, female with a history of previous treatment and surgery for breast tumor, it is important to research the association and the occurrence of metastasis gastric.

## CONCLUSION

In patients with a previous history of breast cancer showing an endoscopic tumor in the stomach, should be considered the possibility of breast cancer metastasis. Moreover, after diagnosis of a breast cancer metastasis to the stomach, surgical resection should be indicated considering primary tumor control and absence of involvement of other organs.
